# Case report: Coronary atherosclerosis in a patient with long-standing very low LDL-C without lipid-lowering therapy

**DOI:** 10.3389/fcvm.2023.1272944

**Published:** 2023-09-19

**Authors:** Giorgio Mottola, Francine K. Welty, Hamid R. Mojibian, Kamil F. Faridi

**Affiliations:** ^1^ Department of Medicine, Section of Cardiovascular Medicine, Yale School of Medicine, New Haven, CT, United States; ^2^ Division of Cardiology, Beth Israel Deaconess Medical Center and Harvard Medical School, Boston, MA, United States; ^3^ Department of Radiology & Biomedical Imaging, Section of Vascular & Interventional Radiology, Yale School of Medicine, New Haven, CT, United States

**Keywords:** LDL-C, atherosclerosis, familial hypobetalipoproteinemia (FHBL), primordial prevention of CVD, coronary artery disease

## Abstract

**Background:**

ApoB-containing lipoproteins including low-density lipoprotein cholesterol (LDL-C) are necessary for the development of atherosclerosis, and lifelong exposure to low serum levels of LDL-C have been associated with a substantial reduction of cardiovascular risk. Although plaque regression has been observed in patients with serum LDL-C less than 70–80 mg/dl on lipid-lowering therapy, an LDL-C level under which atherosclerosis cannot develop has not been established.

**Case presentation:**

In this case we describe a 60-year-old man with well-controlled diabetes mellitus and hypertension who presented to the hospital after an acute stroke likely due to an atrial myxoma discovered on imaging. A coronary computed tomography angiography scan performed in preparation for the planned surgical myxoma resection revealed an anomalous origin of the right coronary artery as well as evidence of nonobstructive coronary atherosclerosis in the right coronary and non-anomalous left coronary system. Despite not having ever been on any lipid-lowering therapy, this patient was found to have low LDL-C levels (<40 mg/dl) during this admission and on routine laboratory data collected over the prior 16 years. His family history strongly suggested heterozygous familial hypobetalipoproteinemia as a possible diagnosis.

**Conclusions:**

This case illustrates that even long-standing, very low levels of LDL-C may be insufficient to completely prevent atherosclerosis and emphasizes the importance of primordial prevention of all cardiovascular risk factors.

## Introduction

While the biological mechanisms underlying the pathogenesis of atherosclerosis involve numerous factors, apolipoprotein B (ApoB)-containing lipoproteins inclusive of low-density lipoprotein cholesterol (LDL-C) are necessary for its development (1). Mendelian randomization studies and prospective studies of individuals with genetic variants leading to naturally low LDL-C levels have demonstrated that lifelong exposure to low LDL-C results in substantial reductions in risk of atherosclerotic cardiovascular disease (ASCVD) even in the presence of other risk factors (2). Atherosclerosis detected by coronary calcification can also occur in asymptomatic middle-aged adults without other risk factors and at serum LDL-C levels below 100 mg/dl (3). Reduction in atherogenic lipoproteins early in life is therefore central to primary prevention of ASCVD, though the serum levels of ApoB or LDL-C at which atherosclerosis fails to develop has not been firmly established and likely varies among individuals. It has been hypothesized that lifelong LDL-C of less than 30 mg/dl would be sufficient to completely prevent atherosclerosis (4), though studies of patients with established ASCVD have demonstrated plaque regression with serum LDL-C less than 70–80 mg/dl on lipid-lowering therapy (5).

## Case description

A 60-year-old man presented to his local hospital with new-onset weakness, ataxia and paresthesia in the left upper extremity and was found to have an acute ischemic stroke based on MRI imaging showing multiple areas of acute cerebral and cerebellar infarction. A transthoracic echocardiogram was performed which showed a 4.3 × 2.0 cm mass in the left atrium (Supplementary Video S1), consistent with an atrial myxoma which was thought to be the cause of his acute stroke. He was subsequently transferred to a tertiary medical center for further work-up and evaluation for cardiothoracic surgery. Prior to surgery for surgical myxoma resection, inpatient cardiology was consulted for preoperative evaluation. Aside from mild residual weakness, ataxia and paresthesia in his left upper extremity, the patient was asymptomatic and had been physically active. On physical exam, he was noted to have mild weakness of his left upper extremity (4+/5 strength in shoulder abduction) and trace dysmetria of the left hand. His cardiac exam was notable for a low-pitched sound heard early in diastole with no associated murmurs. His physical exam was otherwise unremarkable.

The patient reported being diagnosed with hypertension and type 2 diabetes mellitus approximately 7 years prior to admission. He was currently taking amlodipine and losartan and reported his systolic blood pressure was typically less than 130 mmHg at all recent clinic visits. At the time of diabetes diagnosis, his hemoglobin A1c (HbA1c) was 12.3 and he reported his diet contained high amounts of processed foods, saturated fat, and sugar-sweetened beverages. He was prescribed metformin and subsequently stopped drinking sugar-sweetened beverages, made other healthful changes to his diet and started exercising on a regular basis. He subsequently lost 20 pounds and his HbA1c after one year was markedly improved at 5.0. On admission his HbA1c was 6.2 with a body mass index of 27.9 Kg/m^2^. He was also noted to have hepatic steatosis on an outpatient liver ultrasound prior to admission. He denied ever using tobacco or drugs and rarely consumed alcohol.

On admission the patient's standard lipid panel showed a serum LDL-C of 32 mg/dl and a non-HDL-C of 46 mg/dl. The patient had never been on lipid-lowering therapy and reported being previously told he had naturally very low blood cholesterol levels, which was confirmed on outpatient labs over the prior 16 years (Table 1). He recalled being told his mother and maternal grandmother had very low levels of LDL-C. He had no family history of premature ASCVD. Further testing during his hospitalization revealed an ApoB level of 35 mg/dl, directly measured LDL-C of 48 mg/dl, and lipoprotein(a) of 36 nmol/L.

**Table 1:** Patient laboratory tests for Serum lipids and HbA1c.

Year	Total cholesterol (mg/dl)	Triglycerides (mg/dl)	HDL-C (mg/dl)	Friedewald LDL-C (mg/dl)	Martin-Hopkins LDL-C (mg/dl)	Non-HDL-C (mg/dl)	Direct LDL-C (mg/dl)	HbA1c (%)
2005	106	–	34	–	–	72	–	–*
2010	96	209	35	19	34	61	–	–
2015	108	440	28	–	43	80	–	12.3
2016	86	68	45	27	27	41	–	5.0
2021	91	77	37	37	38	54	–	–
2022**	86	61	40	34	32	46	48	6.3

* Fasting blood glucose in 2005 was reported as 90 mg/dl.

** At time of hospitalization, the patient's serum apolipoprotein B was 35 mg/d. and his lipoprotein(a) was 36 nmol/L.

**Figure 1:**
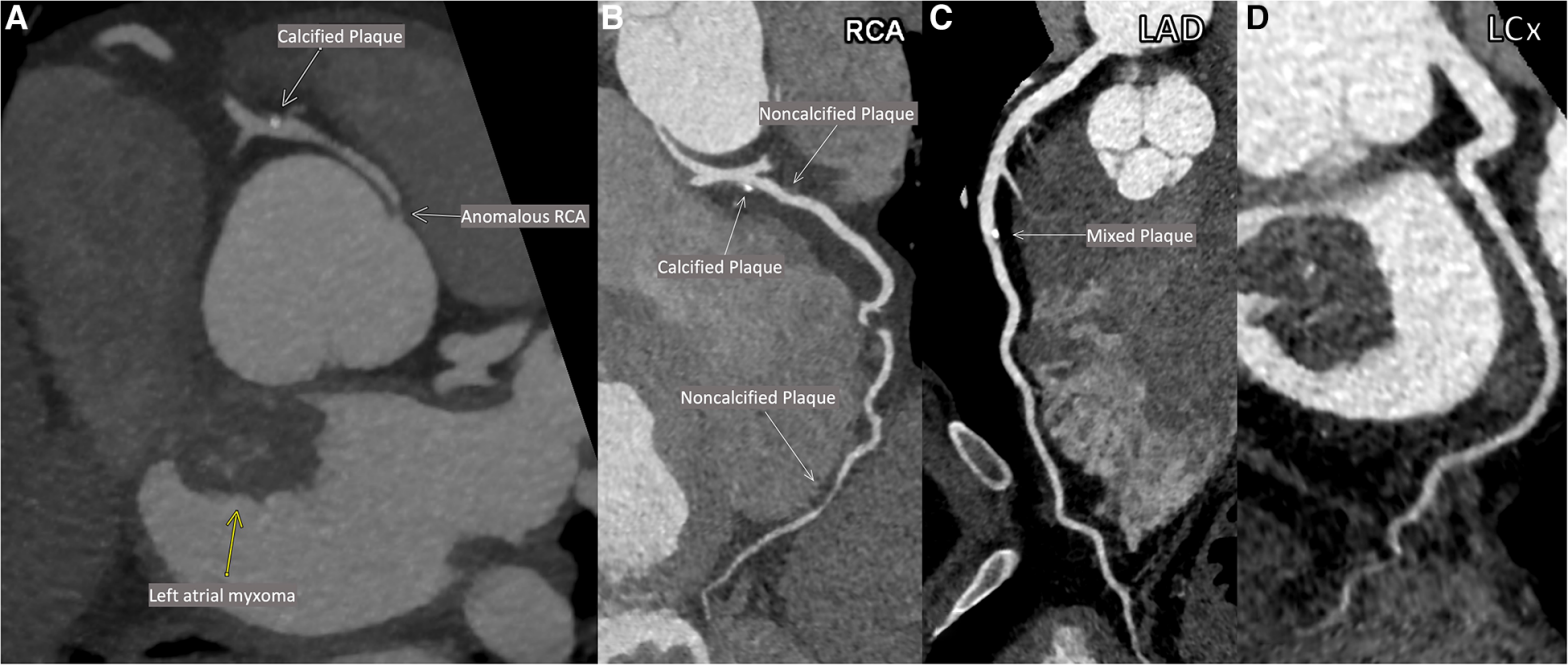
Patient's coronary computed tomographic angiography (CCTA). Anomalous origin of the right coronary artery (RCA; **A**). Multiplanar reconstructions of the RCA (**B**), the left anterior descending artery (LAD; **C**) and the left circumflex artery (LCx; **D**).

As part of pre-operative planning for cardiac surgery, the patient underwent coronary computed tomography angiography (CCTA) which demonstrated an anomalous right coronary artery (RCA; Figures 1A,B) at the origin of the left sinus with mild atherosclerotic plaques in the RCA. Mild atherosclerotic plaques were also noted in the left anterior descending artery (LAD) and left circumflex artery (LCx; Figures 1C,D), with normal LAD and LCx origins. To assess for ischemia due to the anomalous RCA, pharmacologic myocardial perfusion imaging with positron emission tomography was performed which showed a small-sized, mild-intensity, reversible perfusion defect in the apical inferior wall which was thought to be clinically insignificant due to lack of any exertional symptoms.

The patient underwent successful robotic-assisted resection of the left atrial mass (confirmed to be a myxoma on pathology) with no intervention performed to the RCA. He was discharged on post-operative day 4 with rosuvastatin and metoprolol. He was also recommended for consideration of outpatient genetic screening for hypobetalipoproteinemia.

## Discussion

Despite chronic and presumably life-long levels of LDL-C typically less than 40 mg/dl, the patient presented here was found to have mild atherosclerosis in multiple coronary arteries by CCTA. Genetic testing was not performed in this patient, though his family history, long-standing low blood cholesterol levels and hepatic steatosis suggest his low LDL-C is likely due to heterozygous familial hypobetalipoproteinemia, a genetic cause of low LDL-C with a ∼1:1000 prevalence in most populations (6). Patients with familial hypobetalipoproteinemia typically have LDL-C levels between 20 and 50 mg/dl and though the APOB mutations often associated with this condition lead to substantially lower risk of ASCVD (7), prevalence of coronary atherosclerosis specifically in this population has not been well described.

Importantly, this case demonstrates that coronary atherosclerosis can occur even with long-standing LDL-C less than 40 mg/dl when other risk factors such as hypertension and diabetes are present and otherwise well controlled. Reports of atherosclerosis in individuals with lifelong very low LDL-C are extremely rare (8). The patient in this instance did have uncontrolled diabetes mellitus on initial diagnosis with a HbA1c of 12.3 in 2015, and due to lack of regular follow-up it is unclear how long that level of hyperglycemia may have occurred and contributed to plaque development. His serum ApoB was not assessed at that time and his LDL-C and non-HDL-C were higher than on all his other laboratory checks, though still at very low levels. Notably, his diabetes rapidly improved with only lifestyle changes and metformin. At the time of hospitalization, the patient's serum ApoB was concordantly low and his lipoprotein(a) level was also low, and therefore these factors are unlikely to explain development of atherosclerosis. There is little evidence available on whether anomalous coronary artery origin directly contributes to atherosclerosis; it has previously been shown that anomalous right-sided arteries with a retroaortic course may develop atherosclerosis earlier than non-anomalous arteries within the same patient. However, the case patient was also observed to have atherosclerosis in the non-anomalous left coronary system (9). To our knowledge, this is the first case report showing calcified coronary plaque on CCTA in a patient with long-standing LDL-C less than 40–50 mg/dl while not on lipid-lowering therapy.

This case indicates that even lifelong LDL-C at very low levels, as could theoretically be achieved with early initiation of statins and PCSK9 inhibition with medications or gene editing (10–12), may be insufficient to completely prevent atherosclerosis when other risk factors are present. Therefore, primordial prevention of risk factors, rather than only managing them after they have developed, remains critical to reducing prevalence of atherosclerosis and ASCVD across populations. However, atherosclerosis specifically in patients with familial hypobetalipoproteinemia is not well understood, and there may be unique factors that promote development in this population. Given that our observations were made from a single patient, definitive conclusions about coronary plaque development cannot be made from this report. Further study will be needed to determine what specific factors drive development of atherosclerosis in the setting of low LDL-C, including in patients with familial hypobetalipoproteinemia.

## Data Availability

The original contributions presented in the study are included in the article/**Supplementary Material**, further inquiries can be directed to the corresponding author.
