# Supplementary material for: Case report: Coronary atherosclerosis in a patient with long-standing very low LDL-C without lipid-lowering therapy
Source: Front Cardiovasc Med. 2023 Sep 19;10:1272944. doi: 10.3389/fcvm.2023.1272944 (PMC10546007; doi:10.3389/fcvm.2023.1272944)
Supplement: Supplementary file 1 [file Table1.docx]

Table: Patient Laboratory Tests for Serum Lipids and HbA1c

| Year | Total cholesterol (mg/dL) | Triglycerides (mg/dL) | HDL-C (mg/dL) | Friedewald LDL-C (mg/dL) | Martin-Hopkins LDL-C (mg/dL) | Non-HDL-C  (mg/dL) | Direct LDL-C (mg/dl) | HbA1c  (%) |
| --- | --- | --- | --- | --- | --- | --- | --- | --- |
| 2005 | 106 | - | 34 | - | - | 72 | - | -* |
| 2010 | 96 | 209 | 35 | 19 | 34 | 61 | - | - |
| 2015 | 108 | 440 | 28 | - | 43 | 80 | - | 12.3 |
| 2016 | 86 | 68 | 45 | 27 | 27 | 41 | - | 5.0 |
| 2021 | 91 | 77 | 37 | 37 | 38 | 54 | - | - |
| 2022** | 86 | 61 | 40 | 34 | 32 | 46 | 48 | 6.3 |

*Fasting blood glucose in 2005 was reported as 90 mg/dL

**At time of hospitalization, the patient’s serum apolipoprotein B was 35 mg/dL and his lipoprotein(a) was 36 nmol/L
